# The AMA Communication Techniques Survey: A Psychometric Analysis

**DOI:** 10.3928/24748307-20170912-01

**Published:** 2017-11-09

**Authors:** Danielle Walker, Carol Howe

## Abstract

**Background:**

Health care providers are continually seeking to improve patient communication to improve patient outcomes. The American Medical Association (AMA) developed a set of recommended communication practices and an instrument to evaluate health care providers' use of and perceived effectiveness of these techniques.

**Objective:**

The purpose of the study was to assess factor structure of the AMA Communication Techniques Survey using exploratory factor analysis (EFA) and confirmatory factor analysis (CFA) to evaluate reliability of the instrument among nurses and allied health professionals who provide diabetes education.

**Methods:**

A national convenience sample of 522 diabetes educators completed the survey; this sample was split into two subsamples—the EFA (*n* = 302) and CFA (*n* = 220). Of this sample, 60.2% (*n* = 314) were nurses. Factor structure and reliability analysis of the AMA Communication Techniques Survey was conducted.

**Key Results:**

EFA with varimax rotation revealed two internally consistent subscales labeled basic and advanced communication techniques. CFA determined that basic and advanced technique subscales were a good fit for the factors. Basic techniques included items such as speaking slowly and using simple words. Advanced techniques included the Teach-Back method, underlining words in printed materials, and presenting 2 to 3 concepts at a time. The Cronbach's alpha coefficient for the total scale was .81 and .70 (basic subscale) and .74 (advanced subscale), indicating good reliability.

**Conclusions:**

The AMA Communication Techniques Survey appears to be a valid and reliable instrument to examine communication practices of diabetes educators. Additionally, EFA confirms previously hypothesized basic and advanced subscales. However, the factors included in each scale differ from previous conceptualization. **[*Health Literacy Research and Practice*. 2017;1(4):e208–e215.]**

**Plain Language Summary:**

This study established reliability and validity for the American Medical Association Communication Technqiues Survey among diabetes educators. Data support the creation of two separate groups of items: basic and advanced techqniues.

Effective patient provider communication is a constant challenge that all health care providers face. Currently only 12% of adults in the United States have proficient health literacy skills to navigate the health care system and manage their health (U.S. Department of Health and Human Services, Office of Disease Prevention and Health Promotion, 2010). Low health literacy is consistently associated with poorer health status including increased hospitalizations, lower screening rates, and more emergency department use (Berkman, Sheridan, Donahue, Halpern, & Crotty, 2011). Because of these staggering statistics, the Agency for Healthcare and Research recommends the use of health literacy standard precautions to ensure comprehension for all patients regardless of health literacy level by simplifying communication, making the health care setting easier to navigate, and supporting patients to improve their health (Agency for Healthcare Research and Quality, 2013). Healthy People 2020 promotes effective patient provider communication as well, through objectives to improve the health literacy of the population and health care provider communication skills (U.S. Department of Health and Human Services, Offices of Disease Prevention and Health Promotion, 2017). Despite these initiatives, health care providers still need more training on effective communication techniques (Weatherspoon, Horowitz, Kleinman, & Wang, 2015).

## Background

Concentrated efforts to improve communication by providers are currently being integrated into training programs, continuing education courses, and organizational initiatives. As the emphasis on provider communication techniques increased, the American Medical Association (AMA) and health literacy experts developed a set of recommended communication practices. These recommended practices include the use of simple language, the Teach-Back method, speaking slowly, and presenting 2 to 3 concepts at a time (Schwartzberg, Cowett, VanGeest, & Wolf, 2007; Weatherspoon et al., 2015).

From these recommendations, Schwartzberg et al. (2007) created the American Medical Association Communication Techniques Survey (AMA Survey) to describe providers' frequency of use and perceived effectiveness of 14 specific communication techniques. The initial AMA Survey was developed from a review of the literature and an initial pilot survey of physicians to develop and refine items. The AMA Survey consists of two separate scales: frequency of use and perceived effectiveness. The frequency of use scale is a self-reported Likert scale of 1 (never) to 5 (always) for each of the 14 communication techniques with higher scores indicating more routine use of communication techniques. The perceived effectiveness scale consists of self-reports of yes, no, and I don't know for each of the 14 communication techniques. The AMA Survey formats both scales into a single questionnaire. For example, survey respondents are asked (1) how often in the past week did they use Teach-Back and (2) do they believe this technique is effective (Schwartzberg et al., 2007).

Schwartzberg, et al. (2007) used the survey in an initial study with over 300 health care providers, including physicians, pharmacists, and nurses. The 14 communication techniques were conceptually grouped into basic or advanced techniques. For example, simple language, speaking slowly, and handing out printed materials were identified as basic techniques, whereas the Teach-Back technique was considered an advanced technique. Rozier, Horowitz, and Podschun (2011) used the AMA Survey adapting it to dentistry, postulating that the frequency of use scale could be grouped into five domains or subscales: interpersonal communication, Teach-Back method, patient-friendly materials and aids, assistance, and patient-friendly practice. Subsequent studies using the AMA Survey in other dental providers continued to use these groupings of communication techniques. No factor analysis was conducted to confirm either of the suggested subscales or groupings of basic/advanced or the five domains.

Since inception, the instrument has quickly become a common tool to describe communication practices among providers. Researchers have used the AMA Survey in a variety of settings with multiple types of health care providers, including physicians, nurse practitioners, and dental providers. Similarities in findings are noted across the studies. More specifically, health care providers routinely used between 5 and 7 of the recommended communication strategies. Many health care providers, regardless of professional role or setting, reported using simple language and speaking slowly “always” or “most of the time” when communicating with their patients (Horowitz, Clovis, Wang, & Kleinman, 2013; Koo, Horowitz, Radice, Wang, & Kleinman, 2016; McCarthy, Cameron, Courtney, & Vozenilek, 2012; Rozier et al., 2011; Schwartzberg et al., 2007; Weatherspoon et al., 2015). Simple language was the most effective communication strategy (Flynn, Acharya, Schwei, Van Wormer, & Skrzypcak, 2016; Horowitz et al., 2013; Rozier et al., 2011; Schwartzberg et al., 2007; Weatherspoon et al., 2015). Providers who took a communication course outside of typical coursework were significantly more likely to use more communication techniques in practice (Horowitz et al., 2013; Maybury, Horowitz, Wang, & Kleinman, 2013; Schwartzberg et al., 2007; Weatherspoon et al., 2015). Interestingly, some of the studies found that nurses were more likely to report use of the Teach-Back technique compared to other health care professionals (Koo et al., 2016; Schwartzberg et al., 2007).

Although the AMA Survey has been used in many studies (Flynn et al., 2016; Horowitz, et al., 2013; Koo et al., 2016; Maybury et al., 2013; Rozier et al., 2011; Schwartzberg et al., 2007), formal psychometric analyses have been limited. The instrument was developed and reviewed by health literacy experts based on current literature, which provided initial face and content validity. However, no further validation has been conducted. To date, no reliability analyses have been reported with the AMA Survey in any sample, although previous researchers have cited the need (Flynn et al., 2016; Rozier et al., 2011). Additionally, although subscales or groupings have been conceptualized for the frequency of use scale and hypothesized in the literature, no factor analysis has been conducted to confirm these hypotheses.

## Objective

The purpose of the study was to establish reliability and validity of the AMA Survey among nurses and allied health professionals who provide diabetes education. Reliability and validity will be established through assessing the factor structure for the AMA Survey and psychometric evaluation.

## Design

The AMA Survey was used in an initial study that described factors affecting communication practices among nurses and allied health professionals who provided diabetes education. After Institutional Review Board approval at the affiliated university, the survey was distributed at a national convention of diabetes educators. Eligibility to participate in the study required that the participant be a diabetes educator who provided any structured, organized delivery of diabetes education. Those who did not provide diabetes education were excluded from the study. Survey respondents were entered to win a raffle prize of a free registration to the 2017 American Association of Diabetes Educators National Conference. A description of the design, sampling methods, and procedures of the larger study have been described elsewhere (Howe, Walker, & Watts, in press).

## Data Analysis

To examine both exploratory and confirmatory factor analysis using a single data file, the full sample was randomly split into two data sets, one to be used for exploratory and the other for confirmatory analyses. The full sample was used for reliability testing.

### Exploratory Factor Analysis

Exploratory factor analysis (EFA) was conducted on the Likert-scale responses of the frequency of use scale only, as the perceived effectiveness scale does not meet theoretical assumptions of factor analysis. Listwise deletion was used for missing data during EFA. Preliminary analyses were conducted to ensure that responses were reasonably normally distributed and free of outliers. To examine the structure of the items in the frequency of use of scale, an EFA using varimax rotation was conducted on the 14 items. Factors with eigenvalues greater than 1.00 were retained. Items with factor loadings less than .450 and items that loaded on multiple factors were considered criteria for removal from the next round of analyses.

### Confirmatory Factor Analysis

To test the fit of the final model for the frequency of use scale, we conducted confirmatory factor analysis (CFA) using the Lavaan package of R (version 3.1.2). Using the robust maximum likelihood-based estimator, which corrects for non-normality, we used chi-square statistics and four recommended fit indices with agreed-upon cutoff values (Sun, 2005): standardized root-mean-square residual (SRMR), adjusted goodness-of-fit index (AGFI), root-mean-square error of approximation (RMSEA), and comparative fit index (CFI). According to Hu and Bentler (1999), the desirable cutoff values for the SRMR and RMSEA should be .08 and .06, respectively, and the desirable cutoff value for the AGFI and CFI should be .95 and .90, respectively.

## Reliability

Due to differences in data ranges, reliability was assessed separately for the frequency of use and perceived effectiveness scales. Reliability was assessed using Cronbach's alpha for frequency of use scale, and Kuder-Richardson-20 for the perceived effectiveness scale.

## Key Results

### Sample

The final sample of 522 diabetes educators represented a racially and ethnically diverse sample of mostly women health care providers with a mean age of 50.1 years (standard deviation, 12.1). Health professions represented included registered nurses, dieticians, pharmacists, and others **(Table 1)**. Most of the sample practiced as a diabetes educator more than 16 hours per week. The majority (71%) of the sample were certified as diabetes educators, and 88% held a bachelor's degree or higher.

The initial sample was split into two subsamples, in a 60/40 split due to the increased demand of the EFA. The EFA subsample had 302 participants. The CFA subsample had 220 participants. There were no significant differences between subsamples across demographic factors.

## Frequency of Use Scale

Final EFA loadings are reported in **Table 2**. One item, “drawing pictures,” was removed due to a maximum factor loading of .441 and loading on multiple factors. Two items, “following up with a telephone call to check understanding/compliance” (factor loading = .280) and “having patients follow up with office staff to review instructions” (factor loading = .284) were removed due to loading together on a single factor, suggesting that these items tap a different construct than the remaining items in the scale. According to Tabachnick and Fidell (2007), the reliability of a factor with two items depends upon the extent to which the two items are correlated with each other (e.g., *r* = .399, *p* <.001) and relatively uncorrelated with other items. Because these two items were not strongly correlated with each other (*r* < .800) and were also correlated with other items (Pearson Correlation Coefficient ranged from .026–.384), these two items were removed from the final model. The analysis revealed that six items loaded on the first factor and five items loaded on the second factor **(Table 2)**. After evaluating the groupings, factor one was labeled Basic Techniques and factor two was labeled Advanced Techniques. The total variance explained in Basic Techniques was 23.9% and in Advanced Techniques was 23.7%.

**Figure 1.**
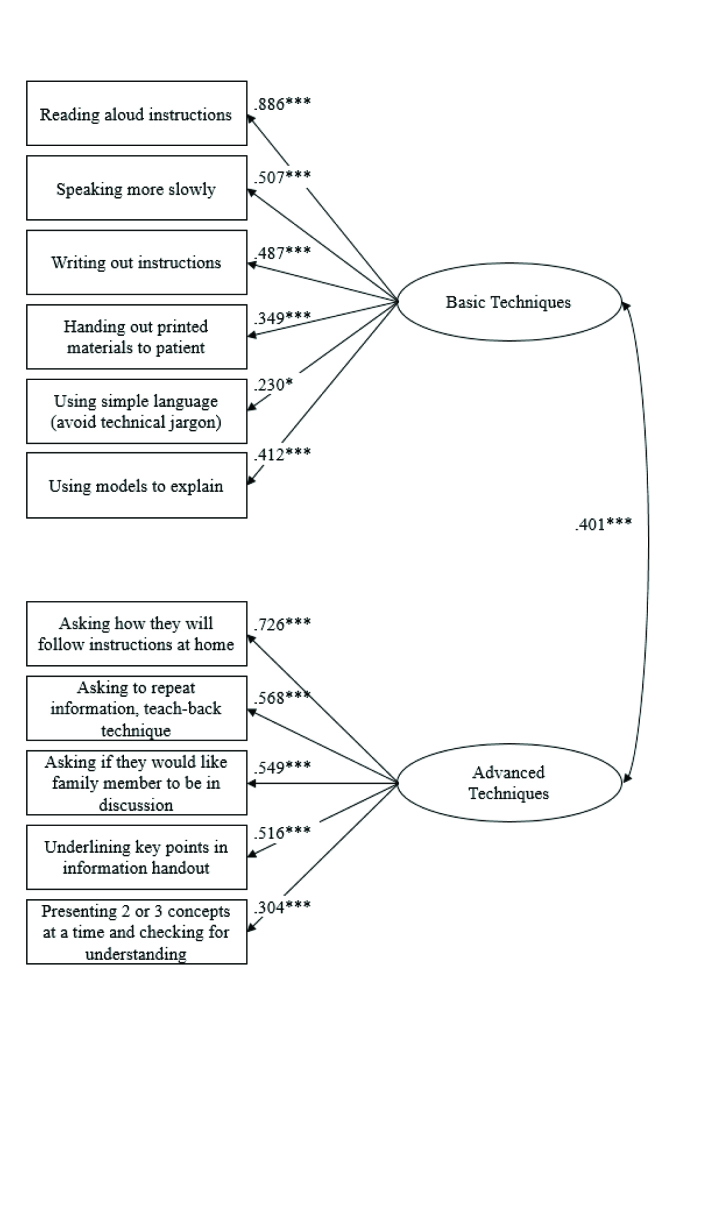
A depiction of the confirmatory factor analysis of the two-factor model for frequency of use scale in the American Medical Association Communication Techniques Survey (*N* = 220). *p < .05; ***p < .001.

As predicted, confirmatory factor analyses confirmed the two-factor model for the frequency of use scale and fit the data satisfactorily, χ^2^(43, *N* = 220) = 57.54, *p* = .068, SRMR = .074, RMSEA = .040, AGFI = .926, CFI = .942. As shown in **Figure 1**, Basic Techniques was positively correlated with Advanced Techniques (latent variable correlation = .401, *p* < .001).

## Reliability

Reliability analyses were conducted on the full sample (*N* = 522) based on the factor structured determined by EFA and CFA. Given that both Basic Techniques and Advanced Techniques subscales displayed strong internal consistency among their two-factor solutions, we computed each factor into a subscale score by computing the mean of the items for each factor **(Table 3)**. Both the Basic and Advanced subscale scores were positively correlated with each other according to a bivariate Pearson's correlation coefficient calculation, *r* = .523, *p* < .001.

Overall reliability for the AMA Survey was in the good to high range. Reliability was conducted for each of the two scales and subscales. Cronbach's alpha for the frequency of use scale demonstrated acceptable reliability (α = .829). Reliability for the Basic (α = .705) and Advanced (α = .738) subscales in the frequency of use scale were also acceptable. A Kuder-Richardson-20 or sum score was computed for perceived effectiveness scale (α = .876) to demonstrate the total number of strategies determined as effective. Descriptions of these scores are shown in **Table 3**.

## Discussion

Originally this survey was designed by health literacy experts within the AMA. Since then, the instrument has been adapted and used with nurses, pharmacists, dentists, and dental hygienists in a variety of settings. This study is the first to use the AMA survey with diabetes educators. The results from this study yielded what previous researchers instinctively knew—this tool assessed communication techniques among health care providers. Factor analysis, both exploratory and confirmatory, confirmed previous discussions of a basic versus advanced factor structure within the frequency of use scale. Internal consistency is acceptable for both the Basic Techniques and Advanced Techniques frequency of use subscales, indicating good reliability. Although the psychometric evaluation indicates the instrument is reliable and CFA indicates the instrument has construct validity, these assumptions should be generalized with caution without confirmation in other sample populations.

Three items were dismissed from the frequency of use scale during the EFA process without negatively affecting reliability results. The suggestion is to remove the items “follow up via phone,” “having office staff follow up,” and “drawing pictures” from the AMA Survey. The items “follow up by phone” and “having office staff follow up” are important aspects of patient care, representing an important concept of follow-up but may not be direct communication skills. EFA supported this idea as these two items initially loaded on a separate, single factor. Although the analysis suggested the removal of “drawing pictures” with an initial low-factor loadings across all factors, analysis supported the inclusion of “using models to explain.” In practice, using models to explain may be similar to drawing pictures. For example, if a diabetes educator draws a MyPlate diagram (U.S. Department of Agriculture, 2017) on a piece of a paper, do they report this technique as using models or drawing pictures? Further investigation using cognitive interviews with providers about the item “drawing pictures” and how it may or may not be related to “using models” is needed before a determination of inclusion or exclusion is made.

A limitation of this study is the convenience sampling methodology. Although the sample was recruited at a national conference allowing for a diverse national sample, convenience sampling limits generalizability. Additionally, the sample population is a limitation to this study. Diabetes educators provide many hours of patient education and may be considered adept at communicating effective patient education. Despite these limitations, we report the first formal reliability and validity data for the AMA Communication Techniques Survey, encouraging further reports of the reliability and validity of this instrument in other populations and settings.

## Conclusions

Developing provider communication skills will continue to be a focus in health care systems to improve health care delivery and patient outcomes. The AMA Communication Techniques Survey appears to be a valid and reliable measure to evaluate communication practices of health care providers, providing assessment to drive improved skills. Although it is important to appreciate this evidence, we must evaluate the reliability and validity of this instrument using other methods.

## Figures and Tables

**Table 1:** Characteristics of the Study Sample of Health Professionals

**Sociodemographic Characteristic**	***n* (%)**

Gender	
Female	496
Male	24

Race/ethnicity	
Caucasian	348 (66.5)
Hispanic	68 (13)
Asian	38 (7.3)
African American	36 (6.9)
Native American	11 (2.1)
Other	22 (4.2)

Licensed professional	
Nurse	314 (60.2)
Dietician	148 (28.4)
Pharmacist	28 (5.4)
Other	32 (6.2)

Education level	
Associate's degree	64 (12.3)
Bachelor's degree	215 (41.2)
Master's degree	192 (36.8)
Doctoral degree	51 (9.8)

Note. Mean age 50.1 years (standard deviation 12.1 years).

**Table 2:** Exploratory Factor Analysis Loadings for Frequency of Use (*N* = 495)

**Factor Name and Item**	**Factor 1**	**Factor 2**

Basic techniques		
Using simple language (avoid technical jargon)	**.477**	.152
Handing out printed materials to patient	**.709**	.148
Speaking more slowly	**.636**	.235
Reading aloud instructions	**.767**	.113
Writing out instructions	**.709**	.148
Using models to explain	**.603**	.152

Advanced techniques		
Presenting two or three concepts at a time and checking for understanding	.306	**.425**
Asking how they will follow instructions at home	.158	**.822**
Asking if they would like family member to be in discussion	.101	**.743**
Asking to repeat information (teach-back technique)	.141	**.784**
Underlining key points in information handout	.422	**.507**

Note. Bold values indicate highest loading. Extraction method used was the principal component analysis. Rotation method used was Varimax with Kaiser normalization. Listwise deletion was used to handle missing data.

**Table 3:** Reliability, Means, and Standard Deviations of Frequency of Use, Basic and Advanced Techniques Subscales, and Perceived Effectiveness

**Scales and Subscales**	**Alpha**	**Number**	**M**	***SD***	**Min**	**Max**

Frequency of use	.829	516	3.87	0.57	2.36	6
Basic techniques	.705	517	4.04	0.63	2.33	8
Advanced techniques	.738	516	4.02	0.67	2.40	6
Perceived effectiveness	.876	343	10.83	4.05	0	18

Note. M = mean; Max = maximum score; Min = minimum score; *SD* = standard deviation. Basic techniques, advanced techniques, and frequency of use scores represent mean scores (Cronbach's alpha are displayed in the table), whereas perceived effectiveness scores represent sum scores (Kuder-Richardson [20 alpha] is displayed in the table).
